# Psychosocial Determinants of Tobacco Use among School Going Adolescents in Delhi, India

**DOI:** 10.1155/2014/170941

**Published:** 2014-11-06

**Authors:** Varun Kumar, Richa Talwar, Neelam Roy, Deepak Raut, Saudan Singh

**Affiliations:** Department of Community Medicine, Vardhman Mahavir Medical College and Safdarjung Hospital, New Delhi 110029, India

## Abstract

*Background.* Tobacco use is one of the major preventable causes of premature death and disease in the world. Many psychosocial factors were found to influence tobacco use. Therefore the present study was designed to determine the role of psychosocial factors associated with tobacco use among school going adolescents in Delhi, India. *Methods.* Cross-sectional study was conducted from February 2013 to September 2013 in four government schools in South district of Delhi, India. The questionnaire contains questions adapted from GYTS (Global Youth Tobacco Survey) to find the prevalence and pattern of tobacco use among adolescents. Data were analyzed using SPSS version 21. *Results.* The prevalence of ever and current tobacco use was found in 16.4% and 13.1%. Current smoking and current tobacco chewing were found in 10.2% and 9.4% students, respectively. The risk of current tobacco use was found to be higher among males (*P* value = 0.000) and in those who got higher pocket money (*P* value = 0.000). Psychosocial factors like lower general self-efficacy and maladjustments with peers, teachers, and schools were also found to be significant predictors of current tobacco use. *Conclusion.* The study has revealed higher prevalence of ever and current tobacco use among adolescent students in Delhi, India.

## 1. Introduction

Tobacco use is one of the major preventable causes of premature death and disease in the world [1]. A disproportionate share of the global tobacco burden falls on developing countries, where 84% of 1.3 billion current smokers reside. Nearly 70% of the world's smokers live in low and middle-income countries [2]. The World Health Organization (WHO) attributes approximately 5 million deaths a year to tobacco. The number is expected to exceed 10 million deaths by 2020, with approximately 70% of these deaths occurring in developing countries [3]. India is the second largest consumer of tobacco in the world. The tobacco situation in India is unique because of a vast spectrum of tobacco products available for smoking as well as smokeless use. The early age of initiation underscores the urgent need to intervene and protect this vulnerable group from falling prey to this addiction. In India alone, nearly 1 in 10 adolescents in the age group 13–15 yr have ever smoked cigarettes and almost half of these report initiating tobacco use before 10 yr of age. Addiction to tobacco and harmful nontobacco products by youth is assuming alarming proportion in India [4].

Recent studies have found that tobacco use is increasing among school children in India and a sizeable proportion of them experiment with drugs quite early in life [5]. Among the youth, late adolescents belonging to 16–19 years age group are particularly vulnerable due to increasing academic pressures, encouragement by peers, lure of popularity, and easy availability. Early initiation of substance abuse is usually associated with a poor prognosis and a lifelong pattern of deceit and irresponsible behavior [6].

A number of factors are found to influence the use of tobacco by adolescents. Some of these are the family history of tobacco use by elders, peer influence, experimentation, and easy access to such products along with personality factors and underlying emotional and psychosocial problems [7]. Lower general self-efficacy and self-esteem, dependency, powerlessness, and social isolation all increase the tendency to any substance use behavior including tobacco use. Social influences to smoke appear to be among the most critical factors in smoking acquisition [8]. Adolescents who reported low levels of parental support, affection, monitoring, and more family control and conflict are prematurely impelled to develop all kinds of antisocial activities including tobacco use [9]. Many studies have reported association between tobacco use and psychosocial determinants [10] and also with lower general self-efficacy [11] among adolescents.

Tobacco companies are aggressively seeking new markets in the developing countries as they are subjected to increased regulation in developed countries [12]. Abundant tobacco production, coupled with weak enforcement of tobacco control measures and easy accessibility and affordability of these products are other factors leading to the rise of the epidemic of tobacco use among adolescents in developing countries [13].

The prevention of tobacco use in young people appears to be the single greatest opportunity for preventing noncommunicable disease in the world today. Adolescents are adopting behavioral patterns that are comparable from country to country [14]. To counteract this effect in India as well as in the rest of developing world, there is an urgent need for good, scientifically sound data about tobacco use pattern that would allow cross-country and within country comparison. Therefore the present study was conducted with the objectives of finding the prevalence and patterns of tobacco use and the role of psychosocial determinants associated with tobacco use among senior secondary school students in Delhi, India.

## 2. Materials and Methods

### 2.1. Study Design

Questionnaire based cross-sectional study was conducted from February 2013 to September 2013 in four government senior secondary schools in South district of Delhi located in the field practice area of the department of Community Medicine, Vardhman Mahavir Medical College (VMMC) and Safdarjung Hospital, New Delhi. All students studying in class 11 and class 12 who were present on the day of visit and agreed to take part in the study were included. Complete enumeration of the study subjects was done.

### 2.2. Study Tool

The questionnaire contains questions adapted from GYTS (Global Youth Tobacco Survey) to find the prevalence and pattern of tobacco use, Schwarzer's General Self-efficacy Scale (GSES) to find the general self-efficacy, and Pareek's Preadolescent Adjustment Scale (PAAS) to find psychosocial maladjustments in five psychosocial domains (Home, School, Teachers, Peers, and General). Even though PAAS is referred to as preadolescent scale, it is widely used in Indian studies among adolescents of all age groups [15]. Bilingual questionnaire, containing both the English and the local language (Hindi) versions, was used.

In GSES, scores were given on Likert scale. The median score of GSES was taken to dichotomize the study subjects into two groups. Those having GSES scores less than the median value were considered to be low in their general self-efficacy and those having scores greater than the median value were considered to be high in their general self-efficacy. PAAS contains 40 questions on “yes” or “no” pattern in five psychosocial domains, Home (9), School (8), Teachers (8), Peers (8), and General (7). High positive score in a domain indicates good adjustment and scores less than “0” indicate maladjustment in that domain.

### 2.3. Data Collection

The questionnaire used in the study was pretested among the adolescent students in different areas and necessary modifications were made to make it more understandable for the students. Prior to data collection, an elaborative briefing about the questionnaire was done to all students in the class.

### 2.4. Data Analysis

Data were analyzed using Statistical Package for Social Sciences (SPSS) version 21. Chi-square test was applied in bivariate analysis with categorical variables. Binary logistic regression analysis with backward elimination was used to determine the independence of associations observed in bivariate analysis by controlling for potential confounding factors. Goodness of fit of the model was tested by Hosmer and Lemeshow test.

### 2.5. Ethical Issues

The study protocol was approved by the Institutional Ethical Review Board of Vardhman Mahavir Medical College (VMMC) and Safdarjung Hospital, New Delhi. Permission for conducting the study was also obtained from the school principals. Informed, written consent was also obtained from the parents or guardians of the school students.

## 3. Results

### 3.1. Sociodemographic Characteristics

Among 962 study subjects, the majority, 675 (70.1%), were of either 16 or 17 years of age. The mean age of the study subjects was 16.88 years (SD = 0.984). Majority were males, 524 (54.5%). 661 (68.7%) of the study subjects belonged to joint family and 759 (78.9%) were Hindu by religion. According to revised Kuppuswamy's socioeconomic classification 2012, most of the study subjects belonged to lower middle class, 422 (43.9%), followed by upper middle class, 399 (41.5%) (Table 1). In majority of the study subjects, the father was working mostly either as a clerk, shop keeper, or farmer, 497 (51.7%), while mothers were house wives, 874 (91%) in most instances.

### 3.2. Ever Tobacco Use

The prevalence of ever tobacco users (who had used tobacco (either smoked or chewed) at least once in his/her lifetime) was found to be 16.4% (95% CI 14.2 to 18.9) in our study. The prevalence of ever tobacco use among male and female students was 20.6% (95% CI 17.4 to 24.3) and 11.4% (95% CI 8.8 to 14.8), respectively. The prevalence of current tobacco users (who had used tobacco (smoked or chewed) at least once in the past 30 days) was found in 13.1% (95% CI 11.11 to 15.38) students whereas the prevalence of current smokers was found to be 10.2% (95% CI 8.42 to 12.27) and current tobacco chewers 9.4% (95% CI 7.67 to 11.37).

Among ever smokers whose prevalence was 15.4% (95% CI 13.2 to 17.8), 95.3% (141/148) smoked cigarettes whereas 4.7% (7/148) smoked hukka. Among ever cigarette smokers, 63.1% (89/141) smoked between 2 and 5 cigarettes per day and median number of cigarettes smoked per day was 2 (IQR 1–3). The prevalence of ever tobacco chewers was 12.5% (95% CI 10.5 to 14.7). Among them, 76.7% (92/120) consumed Ghutka (a manufactured smokeless tobacco product and is a mixture of areca nut, tobacco, and some condiments), 9.2% (11/12) consumed Khaini (consists of roasted tobacco flakes mixed with slaked lime), 8.3% (10/120) consumed Paan Masala (a balanced mixture of betel leaf with lime, areca nut, clove, cardamom, mint, tobacco essence, and other ingredients), and 5.8% (7/120) consumed Zarda (hygienically processed and packed small pieces of tobacco leaves boiled and dried along with slaked lime and spices).

Most of the students (53.3%) initiated tobacco smoking at 13 years of age and 26.4% of students initiated tobacco smoking at 12 years of age with mean age of initiating tobacco smoking being 12.31 years (SD = 1.7). Similarly, 58.1% of students initiated tobacco chewing at 13 years of age followed by 19.4% of students at 12 years. The mean age of initiating tobacco chewing was 12.88 years (SD = 2.1). The median pocket money among ever tobacco users was 300 (IQR 100–900) Indian rupees whereas it was 100 (IQR 50–200) Indian rupees in nontobacco users.

More than three-fourth (76.4%) of the students purchased tobacco products directly from the shop followed by 11.6% of students borrowing them from someone else. Majority of the students (64.1%) had at least one of their family members using tobacco products. Most of them started using tobacco products due to curiosity (41.2%) while 38.5% students used them to relieve their stress and 21.5% started using them due to peer pressure.

### 3.3. General Self-Efficacy and Psychosocial Maladjustments

The median score in Schwarzer's General Self-efficacy Scale (GSES) was 27.5. This value was taken to dichotomize the study subjects into two groups. Those having GSES scores less than the median value were considered to be low in their general self-efficacy and those having scores greater than the median value were considered to be high in their general self-efficacy.

In Pareek's Pre-adolescent Adjustment Scale (PAAS) among 962 students, 121 (12.6%) scored less than “0” in PAAS psychosocial domain “home” and they were found to be maladjusted towards home. Similarly 127 (13.2%) scored less than “0” in PAAS psychosocial domain “school” and found to be school maladjustment, 129 (13.4%) scored less than “0” in PAAS psychosocial domain “teacher” and found to be maladjusted towards teachers, 182 (18.9%) scored less than “0” in PAAS psychosocial domain “peers” and they were found to be in peer maladjustment, and 303 (31.5%) scored less than “0” in PAAS psychosocial domain “general” and they were found to be in general psychosocial maladjustment.

### 3.4. Current Tobacco Use

In bivariate analysis, current tobacco use was found to be more among male students than female students (*P* value = 0.000), students who were getting pocket money of more than 100 Indian rupees (*P* value = 0.000), and in those whose parents use tobacco (*P* value = 0.000). Current tobacco use was also found to be significantly higher in those who were lower in their general self-efficacy (*P* value = 0.000). Home and general psychosocial maladjustment was not found to be significantly associated with current tobacco use among school going adolescents but it was significantly associated with school maladjustment (*P* value = 0.001), teacher maladjustment (*P* value = 0.002), and peer maladjustment (*P* value = 0.025) (Table 2).

## 4. Discussion

The prevalence of ever tobacco use was found to be 16.4% while the prevalence of ever smokers was 15.4% and ever tobacco chewers was 12.5% in the present study. Similarly the prevalence of current tobacco use was found to be 13.2%. The prevalence of current smokers and current tobacco chewers was 10.2% and 9.4%, respectively. These findings were similar to the results obtained in previous studies conducted among school going adolescents aged 15–19 years in Delhi [16]. In the National GYTS study conducted in 2004 in India, the prevalence of ever tobacco use was found to be 25.1% whereas current tobacco use was found to be 17.5% [17].

Tobacco use, especially smoking, is a male-dominated phenomenon among children and adolescents in India unlike the West, where its distribution is equal among both genders. In some countries like China, Fiji, Jordan, and Venezuela, smoking is rather more common among females [7]. In our study, we have found ever tobacco use to be significantly higher among male students (20.6%) than female students (11.4%). Similar results have also been obtained in other studies done among school going adolescents in Kolkata [18]. But recent studies have also shown that tobacco use is on rise among female adolescents in India [16].

The mean age for tobacco use initiation (smoking and chewing) in our study was found to be 12.31 and 12.88 years which were similar to studies from Kathmandu (Nepal) [19], Noida [20], and Kerala (India) [21] where the mean ages of onset were found to be 14.15, 12.4, and 13.2 years, respectively. Early and mid-adolescence are more vulnerable to initiation of tobacco use and hence a targeted intervention is necessary to reduce the tobacco uptake in this age group. Type of family was also found to be an important factor in adolescent tobacco use. In our study, we found that adolescents belonging to nuclear families were 1.5 times (OR = 1.52; 95% CI 1.07 to 2.17) more likely to use tobacco than those belonging to joint families. Similar results have also been reported by a study done in Delhi, India [16]. Adolescents belonging to joint families are under constant adult surveillance which may reduce their risk taking behaviors including tobacco use.

We have found that in our study the likelihood of tobacco use was found to be higher in students belonging to higher socioeconomic class and those getting higher pocket money. A study done in Nepal has also found similar association [3]. Having some amount of money in hand predisposes adolescents to tobacco use by easing access.

The parental tobacco use was reported by 29.9% of adolescents. This result is similar in the median prevalence of 30.6% in a cross-sectional household survey from 26 states of India [22]. Ever tobacco use was found to be 4.3 times (OR = 4.32; 95% CI 3.03 to 6.16) higher in adolescents who had reported parental tobacco use. Tobacco use by family members is likely to influence adolescents who are more likely to perceive tobacco use as a positive and acceptable behavior.

In the present study, the prevalence of ever tobacco use was found to be significantly associated with low general self-efficacy. Fagan et al. in their study among 15–18-year-old adolescents in Boston, USA, found that decreased self-efficacy beliefs increased nicotine dependence [11]. D'Silva and Aminabhavi in their study among adolescents in Goa found that drug addicted adolescents were having lower general self-efficacy [23]. These observations convey the fact that substance use is associated with low general self-efficacy.

In previous studies, smoking status among adolescents has been found to be related to school performance. Those students, who do well in school, have high academic aspirations and are less likely to smoke than those who do not possess these characteristics. The protective effect of academic performance and aspirations on adolescent smoking may reflect beliefs necessary for academic success [10]. In our study, we have found that adolescents who were maladjusted with their schools and teachers were at more risk of using tobacco.

Children and adolescents with anxiety and depression are likely to use tobacco and other drugs, as these have anxiety relieving and mood elevating properties. Furthermore, such children may socially be anxious and feel isolated in a company of peer groups. Peer maladjustment was found to be an important factor in initiating tobacco use among adolescents [24]. Similar findings were obtained in our study where the risk of tobacco use was found to be 1.78 times higher in those who were maladjusted with their peer groups.

Our study is not without limitations. Since data was collected by self-administered questionnaire, both overreporting and underreporting are possible. Recall bias can also occur. The interpretations are restricted to school going late adolescent students only. Further studies are needed that cover the groups of adolescents who are out of school, as the prevalence of health risk behaviors is likely to be higher among such adolescents. Also, detailed analysis of the various psychosocial determinants of adolescent tobacco use was limited by the study being a cross-sectional one. Qualitative research methods like focused group discussions can be utilized in further studies to have in-depth analysis of the reasons for tobacco use among adolescent students.

## 5. Conclusion

The study has revealed that tobacco use is prevalent among adolescent students in Delhi, India. The risk of tobacco use is found to be higher among males and in those students who are getting higher pocket money. Psychosocial factors like lower general self-efficacy and maladjustments with peers, teachers, and schools were also found to be significant predictors of tobacco use.

The results have highlighted the fact that there is an urgent need to take effective steps, in curbing this problem among adolescents. This demands behavioral interventions at several psychosocial environments with which they encounter like home, school, and public places. Awareness programs can be launched and parents, teachers, and peer groups may be involved to educate them about the consequences of tobacco use, and their effectiveness in curbing the problem should be assessed.

## Supplementary Material

General Self-efficacy of the study participants was assessed by Schwarzer's General Self-efficacy Scale (GSES). It contains 10 questions and scores were given on Likert scale. Psychosocial maladjustment was assessed by Pareek's Pre-adolescent Adjustment Scale (PAAS). It contains 40 questions in five different psychosocial domains, Home, School, Teachers, Peers and General.

## Figures and Tables

**Table 1:** Distribution of study participants according to sociodemographic profile (*N* = 962).

S. number	Sociodemographic profile	Number	Percentage
1	Sex		
	Male	524	54.5
	Female	438	45.5

2	Class		
	11	517	53.7
	12	445	46.3

3	Type of family		
	Joint	661	68.7
	Nuclear	301	31.3

4	Religion		
	Hindu	759	78.9
	Muslim	203	21.1

5	Socioeconomic class (Revised Kuppuswamy's classification, 2012)		
	I	42	04.4
	II	399	41.5
	III	422	43.9
	IV	87	09.0
	V	12	01.2

**Table 2:** Distribution of study participants according to current tobacco use (*N* = 962).

Characteristics	Current tobacco user *n* (%)	Nonuser *n* (%)	*P* value
Sex			
Female	30 (6.9)	408 (93.1)	
Male	96 (18.3)	428 (81.7)	0.000
Class			
11	58 (11.2)	459 (88.8)	
12	68 (15.3)	377 (84.7)	0.062
Type of family			
Joint	80 (12.1)	581 (87.9)	
Nuclear	46 (15.3)	255 (84.7)	0.175
Religion			
Hindu	105 (13.8)	654 (86.2)	
Muslim	21 (10.3)	182 (89.7)	0.190
Socioeconomic class			
Low	66 (12.7)	455 (87.3)	
High	60 (13.6)	381 (86.4)	0.667
Pocket money			
<100 INR	28 (5.8)	453 (94.2)	
≥100 INR	98 (20.4)	383 (79.6)	0.000
Parental tobacco intake			
Absent	55 (8.2)	619 (91.8)	
Present	71 (24.6)	217 (75.4)	0.000
General self-efficacy			
High	30 (6.2)	451 (93.8)	
Low	96 (19.9)	385 (80.1)	0.000
Home maladjustment			
Absent	106 (12.6)	735 (87.4)	
Present	20 (16.5)	101 (83.5)	0.231
School maladjustment			
Absent	94 (11.3)	741 (88.7)	
Present	32 (25.2)	95 (74.8)	0.001
Teacher maladjustment			
Absent	95 (11.4)	738 (88.6)	
Present	31 (24.0)	98 (75.0)	0.002
Peer maladjustment			
Absent	93 (11.9)	687 (88.1)	
Present	33 (18.1)	149 (81.9)	0.025
General maladjustment			
Absent	81 (12.0)	578 (88.0)	
Present	45 (14.8)	258 (85.2)	0.741
