# Supplementary material for: Psychosocial Determinants of Tobacco Use among School Going Adolescents in Delhi, India
Source: J Addict. 2014 Nov 6;2014:170941. doi: 10.1155/2014/170941 (PMC4241244; doi:10.1155/2014/170941)
Supplement: Supplementary file 1 — General Self-efficacy of the study participants was assessed by Schwarzer's General Self-efficacy Scale (GSES). It contains 10 questions and scores were given on Likert scale. Psychosocial maladjustment was assessed by Pareek's Pre-adolescent Adjustment Scale (PAAS). It contains 40 questions in five different psychosocial domains, Home, School, Teachers, Peers and General. [file 170941.f1.pdf]

Schwarzer's General Self-efficacy scale

(Never-1, Sometimes-2, Often-3, Always-4)

S.NO	QUESTIONS	NEVER	SOMETIMES	OFTEN	ALWAYS
1	I can manage difficult problems				
2	If someone opposes me, I can find the ways to get what I want				
3	I stick to my aims and goals				
4	I could deal effectively with unexpected events				
5	I know to handle unforeseen situations				
6	I can solve most of my problems				
7	I can rely on my coping abilities				
8	I can find solutions for problems				
9	If I am in trouble, I can usually think of a solution				
10	I can usually handle whatever comes my way				

Pareek's Adolescent Adjustment Scale

S.NO	QUESTIONS	YES	NO
1	My parents get annoyed with me easily		
2	The college is a burden for me		
3	My neighbors are not good people		
4	My parents give me full freedom to play		
5	My teacher encourages me in my studies		
6	I do not want to make friends		
7	My friends do not allow me to do my work		
8	I don't get frightened in the presence of my teachers		
9	The teachers take lot of work from us		
10	I hesitate to speak before others in the class		
11	My friends in the college tease me		
12	My teacher listens to me		
13	My parents take care of my amusements		

14	I love my brothers and sisters at home		
15	My parents interfere with my everyday activities		
16	I feel inferior to others		
17	I am very happy in the company of my friends		
18	I am not friendly with my companions		
19	I want more holidays		
20	Very few friends help me		
21	I don’t like my friends at all		
22	I come to college daily		
23	I don’t feel hesitant in asking questions to the teachers		
24	We have got sufficient freedom in the class		
25	Everybody pays attention to whatever I say		
26	My teacher doesn’t make me understand things		
27	I shall be happy if my teacher is transferred		
28	The rules of the college seems to be senseless to me		
29	I don’t get angry easily		
30	I like my parents very much		
31	My parents don’t allow me to go out alone		
32	Nobody in home understands my difficulties		
33	I like to study very much		
34	I don’t have the habit of quarrelling with others		
35	I don’t like frivolous jokes		
36	Some of my friends are good		
37	I can’t express myself properly		
38	I trust my friends		
39	I forget things in my teacher’s presence		
40	My teacher is very strict		
